# Impact of Advanced HIV Disease on Quality of Life and Mortality in the Era of Combined Antiretroviral Treatment

**DOI:** 10.3390/jcm10040716

**Published:** 2021-02-11

**Authors:** Julia Portilla-Tamarit, Sergio Reus, Irene Portilla, María José Fuster Ruiz-de-Apodaca, Joaquín Portilla

**Affiliations:** 1 Infectious Diseases Unit, Internal Medicine Department, Alicante University General Hospital, 03010 Alicante, Spain; jpt.julia@gmail.com (J.P.-T.); reus_ser@gva.es (S.R.); 2 Alicante Institute for Health and Biomedical Research (ISABIAL), 03010 Alicante, Spain; irene.portilla@ua.es; 3 Department of Clinical Medicine, Miguel Hernandez University, 03207 Elche, Alicante, Spain; 4 Department of Health Psychology, University of Alicante,03690 Alicante, Spain; 5 Psicología Social y de las Organizaciones, Facultad de Psicología, National Distance Learning University (UNED), 28015 Madrid, Spain; gerencial@seisida.net; 6 Spanish Interdisciplinary AIDS Society (SEISIDA), 28036 Madrid, Spain; 7 Spanish AIDS Research Network, Carlos III Health Institute, 28029 Madrid, Spain

**Keywords:** immunodeficiency, HIV/AIDS, mortality, quality of life of healthcare

## Abstract

Currently, AIDS or severe immunodeficiency remains as a challenge for people with HIV (PWHIV) and healthcare providers. Our purpose was to analyze the impact of advanced HIV disease (AHD) on mortality, life expectancy and health-related quality of life (HRQoL). We reviewed cohort studies and meta-analyses conducted in middle- and high-income countries. To analyze HRQoL, we selected studies that reported overall health and/or physical/mental health scores on a validated HRQoL instrument. AIDS diagnosis supposes a higher risk of mortality during the first six months, remaining higher for 48 months. It has been reported that cancer and cardiovascular disease persist as frequent causes of mortality in PWHIV, especially those with previous or current AHD. PWHIV who initiate combination antiretroviral therapy (cART) with CD4 < 200 cells/µL have significantly lower estimated life expectancy than those with higher counts. AHD is associated with lower HRQoL, and a worse physical health or mental health status. AIDS and non-AIDS defining events are significant predictors of a lower HRQoL, especially physical health status. AHD survivors are in risk of mortality and serious comorbidities, needing special clinical attention and preventive programs for associated comorbidities. Their specific needs should be reflected in HIV guidelines.

## 1. Introduction

In the early years of the human immunodeficiency virus (HIV) pandemic, acquired immunodeficiency syndrome (AIDS) was a fatal condition accompanied by severe opportunistic diseases, with physical symptoms such as wasting, diarrhea, fever, and a short-time mortality. In addition, people living with HIV (PWHIV) suffered social isolation, internalized stigma, problems related to drug abuse, depression, anxiety, and other psychosocial conditions [1]. With the advent of high-activity antiretroviral therapy (HAART) in 1996, AIDS mortality decreased dramatically, although serious adverse events secondary to the new antiretroviral drugs began to surface. The HIV population who survived to AIDS, known as long-term survivors [2], frequently suffer a constellation of medical, psychological, and emotional disorders, and low quality of life related to health (HRQoL) [3,4].

Despite improvements in early HIV diagnosis and global efforts to deliver combination antiretroviral therapy (cART), many patients are still at risk of advanced HIV disease (AHD). The Joint United Nations Program on HIV/AIDS (UNAIDS) reported that at the end of 2018, around 770,000 (uncertainty bounds, 570,000–1.1 million) people died from AIDS-related illnesses worldwide [5]. In 2017, the European Centre for Disease Control reported 3130 AIDS diagnoses in the 28 European Union/European Economic Area (EU/EEA) countries, giving a crude rate of 0.7 cases per 100,000 of the population [6].

AHD continues to have a negative impact among some PWHIV, despite antiretroviral therapy [7]. Currently, surviving to AHD can be a real challenge for some PWHIV and their healthcare providers. Our aim is to review the impact of AHD on mortality and HRQoL in PWHIV living in countries with easy access to cART.

## 2. Methodology

Our population of interest was people diagnosed with AHD. We used the stage 3 definition of HIV disease from the Centers for Disease Control and Prevention (CDC), which includes those PWHIV with AIDS or CD4 < 200 cells/µL [8]. For the objectives of this article, we reviewed the scientific literature in PubMed and Google Scholar in English language journals before July 2020, with the condition that most of the study should have been carried out during the 21st century and started after the availability of HAART (1996). We selected cohort studies and meta-analyses conducted in middle- and high-income countries with universal access to cART and to healthcare. To identify independent determinants of mortality, loss of life expectancy, and perceived HRQoL, articles had to report appropriate statistics, especially a multivariate regression analysis on the association between AHD and the corresponding endpoint variables. Moreover, to analyze HRQoL, we selected cross-sectional and longitudinal studies that reported overall health and/or physical/mental health scores on a validated HRQoL instrument.

We searched for the terms “mortality”, “life expectancy”, “cancer”, “cardiovascular disease” and “health-related quality of life”. Each term was crossed with “AIDS diagnosis” or “advanced HIV disease” or “CD4 cells”.

The main reasons to exclude articles were: (1) did not strictly comply with inclusion criteria; (2) did not include patients with AHD or CD4+ lymphocyte counts below 200/µL; and (3) did not include multivariable analysis about the subject of researching. We decided not to review liver disease due to hepatitis C virus (HCV), because the currently available high-active antiviral agents against HCV are decreasing the incidence, morbidity and mortality of serious liver disease in PWHIV.

## 3. Results

### 3.1. Factors Associated to AHD or Progression to AIDS

Studies conducted in high-income countries showed that the populations most likely to present for care with AHD are men, heterosexuals, older people, people who inject drugs, migrants, people suffering socioeconomic inequalities, and people with lower educational levels [9,10].

Factors associated with progression to AHD are social (previous incarceration, non-continuity in HIV care) and immunovirological outcomes (low CD4+ count, poor CD4+ response to therapy, drug resistance). The adjusted risk for progression to AIDS associated with a poor CD4+ recovery (discordant immunovirological response to treatment) was as high as 2.70 (95% CI 1.29–5.66) in some studies [11,12,13,14].

### 3.2. Life Expectancy and Mortality in AHD Patients

Life expectancy in PWHIV on cART has improved worldwide, but an important gap remains compared with the general population [15]. PWHIV at higher risk of mortality in Western countries include people who inject drugs, people living in resource-constrained settings, and people who are highly stigmatized or who face other barriers such as depression and other mental disorders, access to health services, unemployment, family responsibilities, and so on [5,11,15,16].

In our review, we included 14 studies (Table 1): three analyzed life expectancy, and 11 explored mortality associated with AIDS defining-events (ADEs) and non-AIDS defining-events (NADEs). All of them were cohort studies and were mainly conducted in high-income Western countries (Europe and North America), but one included patients from Israel and Argentina.

Cohort studies are quite homogeneous in this regard. Results from longitudinal cohort studies published during the 21st century demonstrate that even on cART, patients with AHD have an increased risk of mortality related with AIDS and non-AIDS events. PWHIV who initiate cART with CD4 < 200 cells/µL have significantly lower estimated life expectancies [16,17,18] and a higher risk of mortality by ADEs and NADEs than those with higher counts [19,20,21,22]. Patients presenting for care with AIDS have a higher risk of mortality during the first six months, and it persists higher during the 48 months after AIDS diagnosis compared with those without previous AIDS [23,24]. After surviving five years with cART, the mortality of patients who started cART with a low baseline CD4 count converged with mortality of patients with intermediate and high baseline CD4 counts [25]. In addition, most of the cohort studies agree that starting cART with higher CD4 counts decrease mortality in PWHIV [26,27,28,29]. These results are independents of gender, age, and type or number of comorbidities (Table 1).

**Table 1:** Impact of advanced HIV disease on life expectancy and mortality.

Reference	Methodology (Cohort Name)	Population	Study Period	Impact of Advanced HIV Disease on Mortality and Life Expectancy
Estimated life expectancy				
Hogg [16]	Cohort study (ART-CC)	Europe/North-America	1996–2005	Expected age at death (years) in 20-year-olds with CD4 count < 100 cells/μL, 100–199 cells/μL and ≥ 200 cells/μL: 32.4 (SE, 1.1), 42.0 (0.62) and 50.4 (0.41), respectively.
May [18]	Cohort study (UK-CHIC)	United Kingdom	2000–2010	Expected age at death (years) at the start of ART in 35-year-olds with CD4 count ≤ 200 cells/μL and 200–349 cells/μL: 71 (95% CI, 68–73) and 78 (74–82), respectively. Expected age of death (years) after 5 years on ART in 35-year-olds with CD4 count < 200 cells/μL and no viral suppression, and CD4 count ≥ 350 cells/μL and viral suppression: 54 (95% CI, 48–61) and 80 (76–83), respectively.
Losina [17]	Cohort study (HIV-Research-Network)	United States	2009 *	Life expectancy in years from age 33 (and additional years lost compared to starting ART with CD4 count ≥ 200 cells/μL) in patients with CD4 count ≤ 200 cells/μL and <50 cells/μL: 18.75 (3.90) and 13.82 (8.83), respectively.
Mortality associated with AIDS				
Montha-luc [24]	Cohort study (ANRS)	France	2003–2009	Risk of death, expressed in aHR (95% CI), in patients presenting with AIDS during first 6 months of follow-up and during months 12 to 48 was 48.3 (28.0–83.5) and 4.8 (3.3–7.0), respectively. Corresponding values among AIDS-free patients with CD4 count ≤ 200 cells/μL were 8.1 (4.5–14.6) and 2.3 (1.6–3.4).
Mocroft [23]	Cohort study (COHERE)	Europe	2000–2011	Increased rate of AIDS/death beyond two years after diagnosis in people presenting for care with AHD in southern Europe (aIRR, 1.38; 95% CI, 1.01–1.88) and in eastern Europe in the first year (aIRR, 6.98; 95% CI, 4.22–11.56).
Mortality associated with CD4 count				
Baker [26]	Cohort study	U.S.A.	1999–2005	Reduction (%) in risk resulting from each increase of 100 cell/μL in CD4 count was 44% (95% CI for HR, 0.50–0.62) for AIDS; 14% (0.77–0.96) for NADEs; 35% (0.59–0.72) for death by any cause; and 30% (0.65–0.75) for the composite outcome of AIDS/non-AIDS event/death.
Marin [27]	Cohort study (CASCADE)	Europe/Canada	1996–2006	Reduction (%) in risk resulting from each increment of 100 cells/μL in the CD4 count was 32% (95% CI, 28–35) for all-cause mortality; 64% (58–69) for ADEs; 33% (18–46) for end-stage liver disease; and 34% (21–45%) for NADC.
Mocroft [28]	Cohort study (EUROSIDA)	Europe/Israel/Argentina	2001–2009	Reduction in risk due to doubling of CD4 count, represented by adjusted IRR (95% CI), was 0.63 (0.60–0.66) for ADEs; 0.80 (0.75–0.85) for NADEs, 0.78 (0.69–0.87) for NADC; 0.70 (0.57–0.80) for ESRD; 0.73 (0.65–0.83) for liver-related events. The reduction in risk of cardiovascular events was not significant (IRR, 0.98; 95% CI, 0.85–1.12).
Young [29]	Cohort study (COHERE)	Europe	1997–2010	HR for new AIDS event or death in people with CD4 < 200/μ/L, 200–350/μ/L and 350–500/μL was 0.35 (95% CI, 0.30–0.40), 0.74 (0.66–0.83) and 0.96 (0.92–0.99), respectively.
May [25]	Cohort study (ART-CC)	Europe/North-America	1996–2013	Adjusting for other prognostic factors and comparing baseline CD4 < 50 cells/µL with 200–349 cells/µL during the first 6 months of ART, MRR was 2.81 (95% CI, 2.12–3.71), declining to 1.59 (1.31–1.92) 3 to 4.9 years after the start of ART. Little evidence that baseline CD4 count was prognostic for mortality after 5 years of cART.
Sobrino-Vegas [20]	Cohort study (CO-RIS)	Spain	2004–2013	aHR for all-cause mortality in people with CD4 count ≤ 200 cells/μL without AIDS and people with AIDS was 5.6 (95% CI, 2.7–11.9) and 22.6 (11.5–44.6), respectively.
Ingle [22]	Cohort study	Europe/U.S.A.	2014 *	CD4 count at death was lowest for those who died of AIDS-related causes, with a median of 48 cells/µL (IQR, 13–140), and highest for those who died of CVD (360; 221–608) and unnatural causes (340; 150–560).
Mortality associated with NADEs				
Zhang [19]	Cohort study (ATHENA)	Netherlands	1998–2012	RR of composite non-AIDS-related endpoint (major CVDs, liver fibrosis/cirrhosis, and non-AIDS-related malignancies) was 4.71 (95% CI, 2.98–7.45) for people with CD4 count < 200 cells/μL, vs. 1.19 (0.82–1.74) for people with CD4 of 350–499 cells/μL; and 2.89 (1.79–4.64) for those with a CDC-C event vs. 1.98 (1.23–3.20) for those with a CDC-B event.
Pettit [21]	Cohort study	Europe/North America	1996–2014	Risk of overall non-AIDS mortality was higher in patients with vs. without ADE (aHR, 2.21; 95% CI, 2.00–2.43).

* year of publication. ADE: AIDS-defining event; aHR: adjusted hazard ratio; aIRR: adjusted incidence rate ratio; ART: antiretroviral therapy; cART: combination antiretroviral therapy; CDC: Centers for Disease Control and Prevention; cHR crude hazard ratio; CI: confidence interval; CVD: cardiovascular disease; ESRD: end-stage renal disease; HR: hazard ratio; IQR: interquartile range; IRR: incidence rate ratio; MHR: mortality hazard ratio; MRR: mortality rate ratio; NADC: non-AIDS defining cancer; NADE: non-AIDS-defining event; RR: relative risk; SE: standard error.

### 3.3. Cancer Incidence and Related Mortality in PWHIV with AHD

Excess of mortality in PWHIV, comparing with the general population of the same sex and age, has been observed in non-AIDS cancer, cardiovascular disease, respiratory diseases, liver diseases, drug abuse, suicide, and other external causes [15]. The more frequent NADEs driving to mortality with previous AIDS or <200 CD4/µL are malignancies, cardiovascular and end-stage liver disease [19,27,28] (Table 1).

Cancers in PWHIV are classified as either AIDS-defining cancers (ADC) or non-AIDS defining cancers (NADC) that includes those related to virus infections (NADCI). We analyzed eight studies on the impact of AHD on cancer development; five were large cohort studies, two were extracted from a Cancer National Registry, and one was a meta-analysis. All of them separately analyzed ADC and NADC in people with previous AIDS or CD4 < 200/cells/µL. Data from these studies demonstrate the strong association between AHD and increased cancer risk in PWHIV (Table 2).

Low CD4 cell count is an independent predictor of developing ADC and NADCI: Kaposi sarcoma, non-Hodgkin lymphoma [30,31,32,33], anal cancer and Hodgkin lymphoma [34,35,36,37]. Data from the ATHENA cohort associate cumulative exposure to CD4 < 200 cells/µL during cART with increased risk of NADCI [37]. Previous AIDS diagnosis is also a risk factor of cancer, compared with those with non-AIDS [36]. Results on the association between AHD and NADC are discordant (Table 2). Some studies show an association between AHD and lung cancer, colorectal and oral cavity/pharynx cancer [31,35], while other studies find no associations [30].

**Table 2:** Impact of advanced HIV disease on cancer incidence.

Reference	Design (Cohort Name)	Population	Study Period	Impact of Advanced HIV Disease on Cancer Incidence
Bedimo [34]	Cancer Registry (U.S. Veterans-Affairs)	U.S.A.	1997–2004.	Median CD4 counts (cells/μL) were significantly lower for PWHIV with NADC (249 vs. 270); anal cancer (156 vs. 270); and Hodgkin lymphoma (217 vs. 269), compared to PWHIV without cancer
Clifford [30]	Cohort study (SHCS)	Switzerland	2005 *	In PWHIV with CD4 count < 100 cells/μL, SIR was 571 (95% CI, 449–716) for KS and 145 (104–197) for NHL. SIR was 25.7 (95% CI, 9.2–56.2) for HL in PWHIV after AIDS diagnosis and 14.9 (7.7–26.1) before diagnosis. For all NADC combined, SIRs before and after AIDS diagnosis were similar.
Silverberg [31]	Cohort Study (Kaiser)	U.S.A.	1996–2008	In HIV-infected people with lower recent CD4 counts vs. non-infected people, there were higher RRs for KS (*p* < 0.001), NHL (*p* < 0.001), HL (*p* < 0.001), anal cancer (*p* = 0.005) and colorectal cancer (*p* = 0.028). RR in HIV-infected people with CD4 count < 200 cells/μL vs. noninfected people was 91.5 (95% CI, 48.0–174.5) for anal cancer and 55.3 (31.3–97.9) for HL. For lung, colorectal and oral cavity/pharynx cancer, RRs were only elevated for HIV-infected individuals with CD4 count < 200 cells/μL.
Chiu [32]	Cancer Registry (British Columbia)	Canada	1996–2008	Independent factors against the development of NADC include the use of triple cART compared to mono-/dual-ART (aOR, 0.64; 95% CI, 0.43–0.95); and higher nadir CD4 counts (aOR, 0.61; 95% CI, 0.41–0.93 for CD4 count ≥ 200 cells/μL).
Kesselring [37]	Cohort study (ATHENA)	Netherlands	1996–2009	Cumulative exposure to CD4 count < 200 cells/μL results in higher risk of NADC (HR, 1.12; range, 1.03–1.22 for each additional year of exposure). In stratified analyses, cumulative exposure to CD4 count < 200 cells/μL was associated with malignancies caused by infections (HR 1.16; range 1.03–1.31) but not with other types of cancers.
Patel [35]	Cohort Study (HOS)	U.S.A.	1996–2010	Factors associated with increased mortality for ADC are lower nadir CD4 counts and HIV VL ≥ 400 copies/mL, with respective HRs of 3.02 (95% CI, 1.39–6.59) and 1.72 (1.01–2.94). Factors associated with increased mortality for NADCNI are lower nadir CD4 counts and detectable HIV VL, with respective HRs of 1.77 (95% CI, 1.07–2.94) and 1.96 (1.18–3.24).
Shiels [36]	Meta-analysis	Multi-site	2007–2009	Cancer incidence in patients with previous AIDS diagnosis compared with those with non-AIDS, expressed as SIR (95% CI), was 8.02 (3.52–18.25) for leukemia, 2.77 (1.43–5.37) for HL, 3.01 (1.69–5.38) for lung cancer, and 3.17 (1.42–7.09) for all cancers combined.
Prosperi [33]	Cohort study (ICONA)	Italy	2010 *	Adjusted RH for ADC and NADC, respectively, by CD4 count: ○ ≤ 50 cells/μL: 20.27 (95% CI, 11.95–34.37; *p* < 0.001) and 2.44 (95% CI, 0.76–7.83; *p* = 0.1) ○ 50–200 cells/μL: 6.48 (95% CI, 3.98–10.57; *p* < 0.001 and 2.76 (95% CI, 1.35–5.65; *p* = 0,006) ○ 200–350 cells/μL: 1.73 (95% CI, 0.99–3.04; *p* = 0.054) and 1.69 (95% CI, 0.9–3.2; *p* = 0.1)

* Year of publication. ADC: AIDS-defining cancer; aOR: adjusted odds ratio; cART: combined antiretroviral therapy; CI: confidence interval; HL: Hodgkin lymphoma; HR: hazard ratio; KS: Kaposi sarcoma; NADC: non-AIDS-defining cancer; NADCNI: non-AIDS-defining noninfection-related cancer; NHL: non-Hodgkin lymphoma; PWHIV: people living with HIV; RR: relative risk; SIR: standardized incidence ratio; VL: viral load.

Regarding mortality, Patel et al. [35] from the HIV Outpatient Study (HOPS) cohort reported that factors associated with all-cause mortality among persons with ADC were a nadir CD4 cell count < 200 cells/mm^3^ and HIV RNA ≥ 400 copies/mL. Among persons with NADCI, no associated factors were identified. Among persons with NADC, factors associated with increased mortality were older age at cancer diagnosis, non-white race, nadir CD4 cell count < 200 cells/mm^3^, viral load ≥ 400 copies/mL, and prior or current history of tobacco use.

### 3.4. Cardiovascular Disease-Related Mortality in PWHIV with AHD

The effect of AHD on the pathogenesis of cardiovascular disease (CVD) is not well established. Traditional cardiovascular risk factors seem to play the main role in this condition. However, some data suggest that AHD may influence the incidence and outcome of CVD and strokes. Authors from the NA-ACCORD cohort analyzed the incidence of type 1 (atherothrombotic) myocardial infarction (T1MI) and risk attributable to traditional and HIV-specific factors. The multivariable analysis showed that lower CD4 counts were significatively associated with higher risk T1MI. The adjusted incidence rate ratios (aIRR) for CD4 counts < 100 cells/μL were: 2.19 (95% CI: 1.44–3.33); 100–199 cells/μL: 1.60 (95% CI: 1.09–2.34); and 200–349 cells/μL: 1.37 (95% CI: 1.01–1.86) [38]. In a systematic review and meta-analysis, Eyawo et al. [39] found that CD4 count < 200 cells/μL was associated with higher MI risk compared with ≥200 cells/μL in three studies and HIV VL ≥ 100,000 copies/mL was associated with increased MI risk compared with <100,000 copies/mL in two studies. However, Feinstein et al. reported that significant mortality predictors for T1MI in PWHIV were only high HIV viral load (HIV-VL), renal dysfunction, and older age; and for a type 2 (supply-demand mismatch) MI, low body-mass index and detectable HIV [40].

In a nationwide inpatient sample from the United States, PWHIV with previous AIDS were significantly more likely than uninfected patients to die during hospitalization after admission for MI (OR: 3.03; 95% CI, 1.71–5.38), or stroke (OR: 2.59; 95% CI, 1.97–3.4) [41]. In the same way, in a retrospective cohort study about the outcomes of MI and cardiogenic shock in PWHIV, AIDS was also associated with higher in-hospital mortality compared with HIV people without previous AIDS (28.8% vs. 21.1%; aOR: 4.12 [95% CI: 1.89–9.00]) [42].

The authors of a Taiwanese study reported cytomegalovirus end-organ disease as an independent risk factor for incident all-cause stroke, and particularly ischemic stroke, in PWHIV [43]. Lastly, data from a meta-analysis including 724 cases of intracerebral hemorrhage linked AIDS diagnosis and low CD4 with higher incidence of this event [44].

All these data suggest the high vulnerability and risk of dying after an AHD diagnosis. Mortality related with NADEs could be prevented with the treatment of traditional risk factors such as smoking, elevated total cholesterol, hypertension, and chronic HCV infection [45]. Exhaustive screening of traditional risk factors is necessary to reduce mortality in PWHIV with previous or current AHD.

### 3.5. AHD and Health-Related Quality of Life

We reviewed 11 articles that analyzed the impact of AHD on HRQoL and that complied with the inclusion criteria (Table 3). These articles were heterogeneous, with different populations and different study designs. They used a variety in measurement tools, including generic and HIV-specific ones. Generic HRQoL measures are those applicable across types and severities of disease, across different medical treatments or health interventions, and demographic and cultural subgroups. Disease-specific measures are those that assess specific diagnosis groups or patient populations such as PWHIV. Six of the reviewed articles were cross-sectional studies, and five had a longitudinal design. All studies were conducted in high-income countries, mostly in North America. Sample sizes ranged from 744 to 2508 PWHIV in the cross-sectional studies, and from 265 to 1000 PWHIV in the longitudinal studies.

All the studies found an association between CD4 count and HRQoL. Some associated low CD4 counts with lower overall HRQoL scores or self-perception of health [46,47,48], while others linked low CD4 count to worse physical health status [49] or mental health status [50,51]. A cross-sectional study using a multidimensional HIV-specific HRQoL measure showed that PWHIV with the lowest CD4 counts had the lowest scores in several HRQoL domains, including physical health status [48]. Longitudinal studies found that lower CD4 count predicted worse physical health status and worse mental health status [52,53,54,55,56].

Some cross-sectional studies [50,51] and longitudinal studies [52,54,56] found that AIDS defining events impaired HRQoL.

Some studies associate worse HRQoL with other variables that are potentially related to HIV disease progression, such as HCV coinfection [46], cART adverse events [50,51,52,54] and medical comorbidities (including HIV-related symptoms and non-AIDS comorbidities) [49,55,56].

In addition to clinical and biological markers, the studies we reviewed consistently associated several socio-demographic and psychosocial factors with worse HRQoL in PWHIV. These factors included ageing, housing, depression, social support, employment, and HIV-related stigma.

**Table 3:** Impact of advanced HIV disease on health-related quality of life. Cross-sectional and longitudinal studies.

Reference (Cohort Name)	No. Patients (Age in Years)	Location	Study Period	HRQoL Questionnaires	Impact of Advanced HIV Disease on HRQoL	Other Factors Associated with HRQoL
Cross-sectional studies						
Aden [46] (WHIS)	*n* = 2508 (Not reported)	U.S.A.	1994–2006	SF-6D	Women with HIV with CD4 count ≤ 200 cells/μL ^2^ had lower mean HRQoL scores than women with HIV with CD4 > 200 cells/μL and women without HIV (*p* < 0.01). Women with HIV with detectable viral load had lower HRQoL scores than those with undetectable viral load (*p* < 0.01). In the multivariate analysis, chronic HCV (*p* < 0.01), and low CD4 count (*p* < 0.01) were independently associated with lower HRQoL	HRQoL scores declined with increasing age and decreasing level of education. Current use of illicit drugs was associated with lower HRQoL scores
Preau [50] (ANRS)	*n* = 2235 (mean, 42.5; SD, 9.4)	France	2003	MOS SF-36	Detectable HIV-VL ^2^ (*p* = 0.02), AIDS-defining events (*p* = 0.003) and adverse HIV treatment reactions (*p* < 0.0001) were associated with low PHS. Low CD4 cell count ^2^ (*p* = 0.0007) and adverse HIV treatment reactions (*p* < 0.0001) were associated with low MHS.	Low PHS was associated with older age, absence of a stable relationship, and uncomfortable housing conditions. Financial difficulties, disclosure of HIV status, anxiety and depression, HIV-related stigma, and use of illicit drugs were associated with both poor PHS and MHS.
Fumaz [51]	*n* = 744 (median, 44; IQR, 37–48)	Spain	2010–2011	MOS-HIV	Variables associated with PHS in men were absence of SAEs (*p* < 0.01) and past OI (*p* < 0.01); and in women, years since HIV diagnosis (*p* < 0.01), absence of SAEs (*p* < 0.01), and past OI (*p* < 0.01). Better MHS was related to higher CD4 count ^1^ (*p* = 0.02) and absence of SAEs (*p* = 0.04) in women with HIV. Men and women coincided in the absence of past OI being related to better MHS (*p* < 0.01).	Employment and HIV not acquired through the injection route was related to better PHS and MHS in men with HIV. Having a stable partner was related only to better MHS in men.
Emuren [49] (NHS)	*n* = 1668 (median, 40; IQR, 32.0–47.0)	U.S.A.	2006–2010	SF-36	CD4 count < 200 cells/μL ^2^ (*p* < 0.0001), AIDS diagnosis (*p =* 0.0009), and medical comorbidities (*p* < 0.0001) were associated with lower PHS. CD4 count < 200 cells/μL ^2^ (*p* < 0.05) was associated with lower MHS.	Being married, lower military rank and ageing were associated with lower PHS. Increasing age and being African American were associated with higher MHS. Mental comorbidities were associated with both lower PHS and MHS.
Fuster [48]	*n* = 1462 (mean, 45.0; SD, 10.2)	Spain	2016–2017	WHOQOL-HIV-BREF	Participants with CD4 count < 200 cells/μL ^1^ had the lowest scores in the following dimensions of HRQoL: general health (*p* < 0.0001), physical health (*p* = 0.004), level of independence (*p* < 0.0001) and environmental health (*p* = 0.005).	Being female, being heterosexual, having low socioeconomic and educational status, having acquired HIV through an injection route, and living more years with HIV were related to poorer HRQoL.
Venturini [47]	*n* = 943 (mean, 50.9; SD, 9.3)	Italy	2015	EQ-5D-3L EQ-VAS	Patients with CD4 count < 200 cells/μL ^2^ compared with > 500 cells/μL had a lower score in the EQ-VAS (*p* = 0.02).	Older age, HCV coinfection, education level and hospitalization due to HIV were associated with a low perception of health (EQ-VAS).
Longitudinal Studies						
Protopopescu [52] (ANRS)	*n* = 1000 (mean, 37.1; SD, 0.3)	France	1997–2000	MOS SF-36	CD4 deficiency and number of self-reported side effects were negatively associated with PHS (*p* < 0.0001 for both variables in both random and joint effect models) and MHS (*p* = 0.021 in random effect model and *p* = 0.018 in the joint model for CD4 count and *p* < 0.0001 in both models for side effects) over 5 years after HAART initiation. Clinical stage of AIDS (*p* < 0.0001 for both random effects and joint model and HCV coinfection were negative predictors of PHS (*p* = 0.047 for both models).	Low age, high education level and being MSM were associated with better PHS. Having a stable partner and HIV infection not acquired through the injection route were associated with higher MHS. Comfortable housing conditions were positively associated with PHS and MHS.
Nieuwkerk [53] (ATHENA)	*n* = 265 (mean, 40.1; SD, 8.7)	Netherlands	1998–2005	MOS-HIV	Patients who started HAART with <200 CD4 cells/μL ^2^ had significantly worse baseline PHS than those who started with >200 CD4 cells/μL. These patients showed a significantly greater improvement in PHS than those who started HAART with higher CD4 count (>200 cells/μL)	
Anis [54] (OPTIMA clinical trial)	*n* = 368 (mean, 48.0; SD, 8.5)	U.S.A., U.K., Canada	2001–2007	MOS-HIV, EQ-5D	ADEs and SAEs ^2^ had significant negative impacts on HRQoL in the multivariate linear regression model. ADEs had a more persistent effect on HRQoL than SAEs, with a larger magnitude of effect across all instruments. Increasing CD4 count was significantly associated with increasing HRQoL scores (*p* < 0.01 for PHS and MHS). Improvements in HIV-VL had significant positive impacts on HRQoL, although the magnitude was smaller than that of CD4 increase (*p* < 0.05 for PHS, *p* < 0.01 for MHS).	
Liu [55] (MACS)	*n* = 636 (median, 43.3; IQR, 39.0–48.2)	U.S.A.	2001–2004	SF-36	Significant indicators of worse PHS were lower CD4 counts ^2^, taking OI medications (*p* < 0.05), increasing number of HIV-related symptoms (*p* < 0.0001) and non-AIDS comorbidities (*p* < 0.0001). An AIDS diagnosis by itself was no longer significantly related to PHS after controlling for variables such as HIV symptoms and the current use of OI medications.	Older age, lower socioeconomic status, fewer male sexual partners, and no alcohol drinking were significant predictors of lower PHS scores. Use of recreational drugs, interruption of ART in the past six months, and low social support were significant predictors of poor MHS.
Emuren [56]	*n* = 812 (median, 42; IQR, 37.0–47.0)	U.S.A.	2006–2010	SF-36	CD4 count < 200 cells/μL ^2^ predicted both PHS (*p* = 0.0008) and MHS (*p* = 0.0003). Medical comorbidities (*p* < 0.0001), and ADE (*p* < 0.0001) were also significant predictors of PHS. Patients with medical comorbidities experienced a yearly improvement in PHS.	Ageing led to a longitudinal reduction in PHS. Mental comorbidities were associated with both PHS and MHS.

ADE: AIDS-defining event; EQ-5D: EuroQol 5-dimension quality of life instrument; EQ-5D-3L: EuroQol 5-dimension-3-level quality of life instrument; HAART: highly active antiretroviral therapy; HCV: hepatitis C virus; HIV-VL: HIV viral load; HRQoL: health-related quality of life; MHS: mental health status; MOS-HIV-30: Medical Outcomes Study HIV Health Survey; MSM: men who have sex with men; OI: opportunistic infections; PHS: physical health status; SAE: serious adverse event; SD: standard deviation; SF-12: 12-Item Short-Form Health Survey; SF-36: 36-Item Short Form Health Survey; SF-6D: 6-Dimension Short Form Health Survey; VAS: visual analogue scale; WHOQOL-HIV-BREF: World Health Organization quality of life survey for HIV patients. Source of biological markers (CD4 cell count, viral load): ^1^ patient self-reported, ^2^ clinical records.

## 4. Discussion and Conclusions

Despite the efficacy and safety of current cART, PWHIV presenting for care with AHD or who progress to AIDS are at higher risk of mortality by new ADEs or NADEs. Longitudinal cohort studies and meta-analyses performed during the cART era demonstrate that AHD is associated with a higher risk of developing ADC and NADC, especially NADCI. Traditional cardiovascular risk factors, exposure to some antiretroviral drugs, and persistent HIV viremia are associated with CVD in PWHIV. However, data from cohort studies suggest that AHD could be associated with stroke, CVD, and related mortality. Patients with previous cytomegalovirus end-organ disease or lower nadir CD4 are at risk of developing ischemic strokes. AHD also leads to worse HRQoL. Most of the studies about HRQoL presented in this review are cross-sectional studies. Unfortunately, longitudinal studies evaluating the quality of life in patients previously diagnosed with AHD are scarce. Of those presented in this review, most include a large number of patients, strengthening the results. These studies agree that lower CD4 and previous AIDS diagnosis are associated with lower HRQoL scores, regardless of the questionnaire used or other known variables associated with worse HRQoL. We need more longitudinal studies that analyze the HRQoL for longer periods of time after an AHD diagnosis.

Antiretroviral Treatment guidelines from the World Health Organization (WHO) [57] and from the British HIV Association (BHIVA) [58] consider the AHD as a special issue, but both guidelines focus their interest on the treatment and prevention of opportunist diseases. Our study highlights the need to define AHD as a special state in the spectrum of HIV disease. The high incidence of NADEs and mortality found in AHD patients could be lowered with interventions on traditional risk factors; hence, the importance of screening for risk factors, improving prevention, and creating sustainable care models to implement these interventions during follow-up. We need more longitudinal studies that analyze the HRQoL for longer periods of time after an AHD diagnosis. In our opinion, HIV guidelines should consider AHD patients as a special population whose needs are different from PWHIV who present for care soon after HIV infection.

## Data Availability

Not applicable.
